# High Salt Induces a Delayed Activation of Human Neutrophils

**DOI:** 10.3389/fimmu.2022.831844

**Published:** 2022-06-03

**Authors:** Ignacio Mazzitelli, Lucía Bleichmar, Claudia Melucci, Pehuén Pereyra Gerber, Agustina Toscanini, María Luján Cuestas, Fernando Erra Diaz, Jorge Geffner

**Affiliations:** ^1^ Instituto de Investigaciones Biomédicas en Retrovirus y SIDA (INBIRS), Consejo Nacional de Investigaciones Cientìficas y Tecnològicas (CONICET), Facultad de Medicina, Universidad de Buenos Aires, Buenos Aires, Argentina; ^2^ Cambridge Institute for Therapeutic Immunology and Infectious Disease (CITIID), Jeffrey Cheah Biomedical Centre, University of Cambridge, Cambridge, United Kingdom; ^3^ Microbiología y Parasitología Médica Instituto de Investigaciones en Microbiología y Parasitología Médica (IMPAM), Consejo Nacional de Investigaciones Cientìficas y Tecnològicas (CONICET), Facultad de Medicina, Universidad de Buenos Aires, Buenos Aires, Argentina

**Keywords:** inflammation, IL-8 (CXCL8), oxygen reactive intermediates, sodium chloride, innate immunity

## Abstract

High salt (NaCl) concentrations are found in a number of tissues under physiological and pathological conditions. Here, we analyzed the effects induced by high salt on the function of human neutrophils. The culture of neutrophils in medium supplemented with high salt (50 mM NaCl) for short periods (30-120 min) inhibited the ability of conventional agonists to induce the production of IL-8 and the activation of respiratory burst. By contrast, exposure to high salt for longer periods (6-18 h) resulted in the activation of neutrophils revealed by the production of high levels of IL-8, the activation of the respiratory burst, and a marked synergistic effect on the production of TNF-α induced by LPS. Increasing osmolarity of the culture medium by the addition of sorbitol or mannitol (100 mM) was shown to be completely unable to stimulate neutrophil responses, suggesting that high sodium but not an increased osmolarity mediates the activation on neutrophils responses. A similar biphasic effect was observed when the function of monocytes was analyzed. Short term exposure to high salt suppressed IL-8 and TNF-α production induced by LPS while culture for longer periods triggered the production of IL-8 but not TNF-α in the absence of LPS stimulation. Contradictory results have been published regarding how high salt modulates neutrophil function. Our results suggest that the modulation of neutrophil function by high salt is strongly dependent on the exposure time.

## Introduction

Neutrophils are the first cells recruited into sites of inflammation or infection, where they play a central role by destroying microbes through different mechanisms, such as phagocytosis, oxidative burst, release of antimicrobial proteins and NETs (1, 2). Once recruited, neutrophil function can be modulated by different biological agents such as pathogen-associated molecular patterns (PAMPs), damage-associated molecular patterns (DAMPs) and cytokines. The influence exerted by physicochemical changes in the environment associated with inflammatory processes, such as sodium accumulation has not been clearly defined.

Tissue accumulation of sodium is not an unusual condition. Large amounts of sodium can be stored in the skin as a consequence of a high salt diet (3). Sodium can be stored in a nonosmotic manner by interacting with glycosaminoglycans such as chondroitin sulfate and hyaluronan as well as in a free manner (4, 5). Direct measurement of sodium content revealed that high salt is a characteristic feature not only in the skin, but also in inflamed tissues (6–8), lymphoid organs (9) and the tumor microenvironment (10, 11).

High-salt intake induces a chronic low-grade inflammation promoting not only hypertension (12), kidney damage (13), and cardiovascular disease (14) but also a more severe outcome in a number of inflammatory diseases (15–18). This association appears to be related, at least in part, to the ability of sodium to modulate the function of either innate and adaptive immune cells. Two major targets of the immunomodulatory actions exerted by sodium have been defined; CD4+ T cells and macrophages. Acting on CD4+ T cells it favors the differentiation of naïve T cells into a TH17 profile (15, 19) while strongly impairs the function of regulatory T cells (16). Acting on macrophages, sodium not only promotes the activation of a pro-inflammatory M1 profile (20, 21), but also inhibits the activation of macrophages in an alternative profile decreasing their ability to suppress the function of effector T cells (22).

In contrast with the observations made in macrophages and T cells, the effect of high salt on neutrophil function remains poorly defined. It is generally assumed that high salt suppresses neutrophil function. It has been reported that high salt prevents the up-regulation of CD11b/CD18 expression (23), the exocytosis of all four neutrophil granule types (24) and the neutrophil chemotactic response (25). However, contradictory observations have also been published. In fact, different studies have reported that high salt enhances (26) or suppresses neutrophil antibacterial activity (27–29). Similar contrasting findings have been reported regarding the ability of high salt to modulate the production of NETs (29, 30) as well as the release of IL-8 by neutrophils (29, 31).

Here, we re-analyzed the influence exerted by high salt on neutrophil function. We found that it induces a biphasic effect characterized by an early inhibition of neutrophils responses induced by conventional agonists followed by the acquisition of a strong inflammatory profile.

## Materials and Methods

### Reagents

Sodium chloride (S5886), D-Mannitol (M4125), D-Sorbitol (S1876), dihydrorhodamine-123 (D1054), diphenyleneiodonium (DPI) (D2926), LPS from *E. coli* (L2630), fMLP (F3506), carboxyfluorescein succinimidyl ester (CFSE) (21888), and Zymosan from *S. cerevisiae* (Z4250) were obtained from Sigma-Aldrich (St. Louis, MO, USA). SB202190 (10010399) and SB203580 (13067) were obtained from Cayman Chemical Company (Ann Arbor, MI, USA). Brefeldin A (BFA) (BD 555029) was obtained from BD Biosciences (Argentina), and pHrodo-red Zymosan BioParticles (P35364) from Thermo Fischer Scientific (Argentina).

### Isolation of Neutrophils and Monocytes and Culture Conditions

Heparinized human blood samples were obtained from healthy donors who gave written informed consent. Neutrophils were isolated from human blood samples by centrifugation on Ficoll-Paque (GE Healthcare, Argentine) and dextran (Sigma-Aldrich) sedimentation. Contaminating erythrocytes were removed by hypotonic lysis. After washing, the cell pellets (>98% of neutrophils) were suspended in RPMI-1640 medium (Gibco Invitrogen, Carlsbad, CA, USA) supplemented with 10% heat-inactivated fetal calf serum (FCS), 50 U/ml penicillin, and 50 μg/ml streptomycin (Gibco Invitrogen) (culture medium). Neutrophil preparations were stained with an anti-CD14-PE antibody and analyzed with a BD FACSCanto cytometer and BD FACSDiva software (BD Biosciences, Franklin Lakes, NJ, USA) to guarantee that monocyte contamination was < 0.5%. PBMCs were isolated by centrifugation on Ficoll-Paque, and monocytes were purified from PBMCs by positive selection using anti-CD14-coated magnetic beads according to the manufacturer’s instructions (Miltenyi Biotec, San Diego, CA, USA) (% purity >88%).

Neutrophils (2 x10^6^/ml) or monocytes (1 x10^6^/ml) were cultured for different periods at 37°C in 5% CO_2_ in culture medium supplemented with 25-50 mM of NaCl. The osmolarity of the baseline culture medium was 295 ± 15 mOsm/l. It was increased to values of ~350 to ~400 mOsm by the addition of 25 or 50 mM of NaCl, respectively. To achieve an equivalent osmotic effect to that induced by the addition of 50 mM NaCl, in some experiments the culture medium was supplemented with 100 mM of sorbitol or mannitol. Osmolarity of the culture medium was measured in all cases using a vapor pressure osmometer (Wescor Co, Utah, USA).

### Measurement of Cytokines by ELISA

Neutrophils (2 × 10^6^/ml) or monocytes (1 × 10^6^/ml) were cultured for different periods at 37°C in 5% CO_2_ in culture medium supplemented or not with 25-50 mM of NaCl, in the absence or presence of LPS (100 ng/ml) or zymosan (50 μg/ml). Then, supernatants were harvested and analyzed for the presence of IL-1, TNF-α, IL-6, IL-8, IL-10 or IL-12 by ELISA, according to the manufacturer’s instructions (R&D Systems, Minneapolis, MS, USA).

### Analysis of IL-8 Production by Intracellular Staining And Flow Cytometry

Neutrophils (2 × 10^6^/ml) were cultured for 8 h at 37°C in 5% CO_2_ in culture medium supplemented or not with 25 mM or 50 mM of NaCl. The Golgi transport inhibitor BFA (1 μg/ml) was added during the last 4 h of culture. Cells were then washed with PBS, stained for IL-8 using the BD Cytofix/Cytoperm™ Kit (BD Biosciences, San Jose, CA, USA) according to the manufacturer’s instructions, and analyzed by flow cytometry. Neutrophils activated with LPS (100 ng/ml) were used as a positive control for IL-8 production.

### Analysis of IL-8 Production by Confocal Microscopy

Neutrophils (2 × 10^6^/ml) were suspended in culture medium followed by 30 min adhesion on poly-L-Lysine coated glass coverslips. Attached cells were washed with PBS and were then cultured for 8 h at 37°C in 5% CO2 in culture medium supplemented or not with 50 mM NaCl. The Golgi transport inhibitor BFA (1 μg/ml) was added during the last 4 h of culture. Cells were washed with PBS and fixed with 4% PFA solution at 4°C for 15 min. Afterward, coverslips were treated for 10 min with glycin 0.1M in PBS solution to quench aldehyde group autofluorescence and blocked with 1% BSA solution in PBS. Coverslips were then incubated overnight at 4° with labeled-mAbs directed to CD11b and IL-8 (BD Biosciences) in PBS 1% BSA. The coverslips were mounted with DAPI-Fluoromount-G (SouthernBiotech) and examined under a confocal microscope (ZEISS LSM 900) using a Plan Apochromat 63 × 1.42 NA oil immersion objective.

### Analysis of IL-8 Production in Whole Blood Samples by Flow Cytometry

Fresh whole blood aliquots (200 μl), either untreated or supplemented with 25 or 50 mM NaCl, were cultured at 37°C in a 5% CO2 humidified atmosphere for 8 h. The Golgi transport inhibitor BFA (10 μg/ml) was added during the last 4 h of the culture. Then, erythrocytes were removed by hypotonic lysis. Intracellular IL-8 staining was performed using the BD Cytofix/Cytoperm™ Kit, and cells were analyzed by flow cytometry. Neutrophils and monocytes were gated on the basis of the forward (FSC) and side scatter (SSC) properties, and the negative expression of CD3 and CD19. The high expression of CD66b and the very low expression of CD14 allow to distinguish neutrophils from monocytes.

### Flow Cytometric Determination of Hydrogen Peroxide Production

Generation of hydrogen peroxide was assessed by quantifying the intracellular oxidation of the indicator dye dihydrorhodamine-123 (Molecular Probes, Carlsbad, CA, USA), a membrane-permeable fluorogenic substrate that is oxidized by hydrogen peroxide to the fluorescent compound rhodamine-123. Briefly, neutrophils (2 × 10^6^/ml) were cultured for 30 min, 4 h, 8 h and 18 h at 37 °C in culture medium supplemented or not with 50 mM NaCl. Then, cells were labeled in the same medium for 20 min at 37°C with dihydrorhodamine-123, stimulated or not with fMLP (0.5 μg/ml) or zymosan (50 μg/ml) for 20 min at 37°C, and the oxidation of dihydrorhodamine-123 to rhodamine-123 was analyzed by flow cytometry.

### Analysis of CD11b Expression by Neutrophils

It was performed by flow cytometry. Cells were cultured for 30 min or 8 h in culture medium supplemented or not with 50mM NaCl. Then, cells were washed and labeled for 30 minutes at 4° in culture medium using mAbs directed to CD11b and isotype controls (BD Biosciences).

### Quantitation of Neutrophil Apoptosis by Annexin-V Binding And Flow Cytometry

Annexin-V binding to apoptotic neutrophils was carried out using an apoptosis detection kit (Immunotech, Argentine). Cells were cultured for 30 min, 8 h or 18 h in culture medium supplemented or not with 50mM NaCl. Then, cells were labeled with annexin-V FITC and propidium iodide and analyzed by flow cytometry.

### Zymography

Gelatinase activity was assayed as previously described (32). Briefly, 50 μl of neutrophil supernatants (5 × 10^6^ cells/ml) were mixed with 10 μl of 5× loading buffer (0.25 M Tris pH 6.8, 50% glycerol, 5% SDS, and bromophenol blue crystals) and loaded onto 10% SDS-PAGE gels containing 1 mg/ml gelatin (Sigma-Aldrich). Following electrophoresis, gels were washed using a solution with 50 mM Tris-HCl, pH 7.5, and 2.5% Triton X-100 (buffer A) for 30 min. Then, gels were washed with buffer A supplemented with 5 mM CaCl_2_ and 1 μM ZnCl_2_ for 30 min, and incubated with buffer A supplemented with 10 mM CaCl_2_ and 200 mM NaCl for 48 h at 37 °C, as previously described (32). Gelatinase activity was revealed by staining with 0.5% Coomassie blue. The unstained band indicated the presence of gelatinase activity, and the band position indicated the molecular weights of the enzyme. Images were taken using the UVP BioSpectrum 810 Imaging System (Thermo Fischer Scientific, Argentina), and gelatinolytic band densities were quantified with Image J software.

### Phagocytosis of *C. Albicans* and Zymosan Particles

*Candida albicans* was grown to stationary phase in YPD medium (Sigma-Aldrich) at 30 °C with orbital shaking at 160 rpm. Labeling of *C. albicans* with CFSE (Invitrogen, Carlsbad, CA, USA) was performed by incubating 1 × 10^8^ yeasts with CFSE (0.5 μM) for 1 h at 37 °C. Yeasts were then washed twice in PBS and suspended in culture medium. Neutrophils were labeled with PKH26 red fluorescent cell linker kit (Sigma-Aldrich), according to the manufacturer’s instructions. The phagocytosis assay was performed by incubating PKH26-labeled neutrophils and CFSE-labeled yeasts at a neutrophil/yeast ratio of 1:1, in medium supplemented or not with 50 mM of NaCl during 40 min at 37°C, in the absence or presence of 10% fresh autologous serum. Phagocytosis was then evaluated by flow cytometry. Phagocytosis of Zymosan particles was assessed using pH sensitive Zymosan particles labeled with Red dye, which fluorescence markedly increases as pH decreases, allowing to distinguish endocyted particles from those attached to the cell surface. It was evaluated by flow cytometry.

### Statistical Analysis

When 2 groups were present, normally distributed data were analyzed by 2-sided t test and skewed data were analyzed by Wilcoxon test. For 3 or more groups, One way-ANOVA or Kruskal–Wallis test were used. Normality was assessed by Shapiro–Wilk test. Data analysis was done with GraphPad Prism software version 8 (GraphPad, San Diego, CA, USA). Differences were considered to be statistically significant at p value <0.05.

## Results

### High Salt Modulates IL-8 Production by Neutrophils in a Biphasic Manner

**Figure 1:**
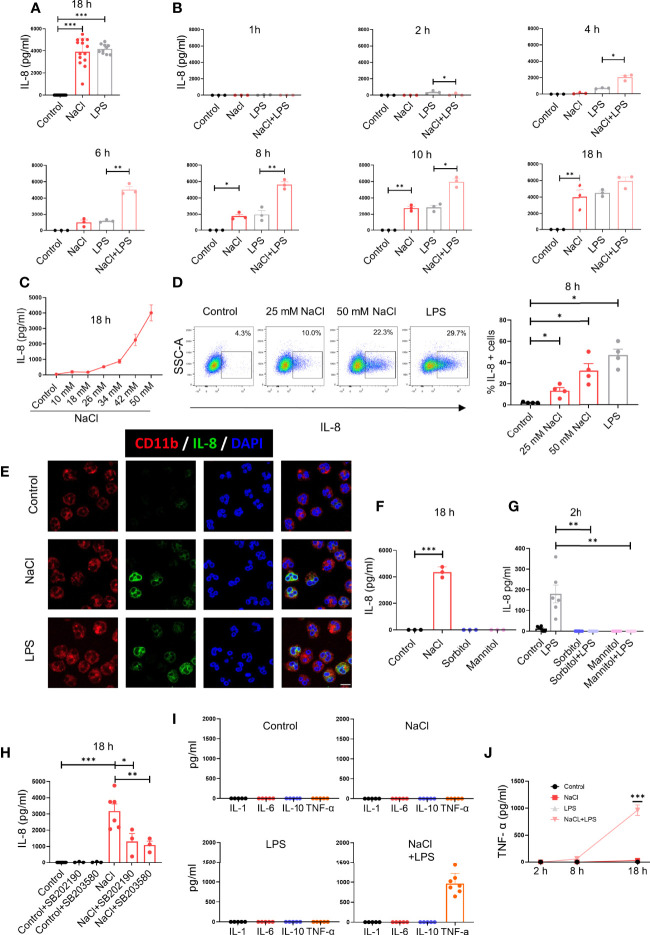
High salt modulates IL-8 production by neutrophils in a biphasic manner. **(A)** Neutrophils (2 x 10^6^/ml) were cultured for 18 h at 37°C in culture medium supplemented, or not, with NaCl 50 mM or LPS (100 ng/ml). IL-8 levels were determined by ELISA in culture supernatants. The mean ± SE from 14 experiments is shown. **(B)** Neutrophils were cultured for different times in culture medium supplemented, or not, with NaCl (50 mM) and/or LPS (100 ng/ml), and IL-8 levels were determined by ELISA in culture supernatants. Results are expressed as the mean ± SE from 3 experiments. **(C)** Neutrophils were cultured for 18 h in culture medium supplemented or not with different concentrations of NaCl. Then, IL-8 levels were determined by ELISA in culture supernatants. Results are expressed as the mean ± SE from 5 experiments. **(D)** Production of IL-8 was evaluated by intracellular staining and flow cytometry after 8 h of culture in culture medium supplemented, or not, with NaCl (25 mM or 50 mM). During the last 4 h of incubation, all samples were treated with the Golgi transport inhibitor BFA (1 μg/ml). Left, a representative experiment is shown. Right, the mean ± SE from 4 experiments is shown. **(E)** Production of IL-8 was evaluated by intracellular staining and confocal microscopy after 8 h of culture in culture medium supplemented. or not, with NaCl 50 mM or LPS (100 ng/ml). A representative experiment (n=3) is shown. **(F)** Neutrophils were cultured for 18 h at 37°C in culture medium supplemented or not with sorbitol or mannitol (100 mM) to increase culture medium osmolarity to values of 400 mOsm. Then, IL-8 production was measured in cell supernatants by ELISA. The mean ± SE from 3 experiments is shown. **(G)** Neutrophils were cultured for 30 min in culture medium supplemented, or not, with sorbitol or mannitol (100 mM) and/or LPS (100 ng/ml), and IL-8 levels were determined by ELISA in culture supernatants. Results are expressed as the mean ± SE from 6 experiments. **(H)** Neutrophils were cultured for 18 h at 37°C in culture medium supplemented or not with NaCl 50 mM, in the absence or presence of the p38 MAPK inhibitors SB202190 or SB353022 (10 μM). Then, IL-8 production was measured in cell supernatants by ELISA. The mean ± SE from 6 experiments is shown. **(I)** Neutrophils (5 x 10^6^/ml) were cultured for 18 h at 37°C in culture medium supplemented or not with NaCl 50 mM, in the absence or presence of LPS (100 ng/ml). Then, the production of IL-1α, IL-6, IL-10, and TNF-α was measured in cell supernatants by ELISA. The mean ± SE from 5-7 experiments is shown. **(J)** Neutrophils were cultured for different times in culture medium supplemented or not with NaCl 50 mM, in the absence or presence of LPS (100 ng/ml). Then, the production of TNF-α was measured in cell supernatants by ELISA. The mean ± SE from 5 experiments is shown. *P <.05, **P <.01, and ***P<.001.

In a first set of experiments we analyzed the influence exerted by high salt on IL-8 production. Neutrophils were cultured for 18 h in medium supplemented or not with NaCl (50 mM) or in the presence of LPS (100 ng/ml), and the concentration of IL-8 in cell supernatants was then analyzed by ELISA. As shown in **Figure 1A**, high salt induced a high production of IL-8 at levels comparable to those induced by LPS. Interestingly kinetic studies revealed that high salt induces opposite effects at early and later times. When evaluated after 2 h of culture, high salt did not induce any production of IL-8 while inhibited IL-8 production induced by LPS. By contrast, when evaluated at 8 and 10 h of culture, high salt not only induced per se the production of IL-8 but significantly increased IL-8 production by LPS-treated neutrophils (**Figure 1B**). Production of IL-8 in response to increased salt concentrations is shown in **Figure 1C**. Similar results were observed when IL-8 production was analyzed by flow cytometry (**Figure 1D**) and confocal microscopy (**Figure 1E**).

To analyze whether high salt or high osmolarity was responsible for the modulation of IL-8 production, further experiments were carried out by increasing osmolarity of the culture medium by the addition of sorbitol or mannitol (100 mM), to reach similar osmolarity levels to those induced by the addition of 50 mM NaCl (400 mOsm). Increasing osmolarity by sorbitol or mannitol did not stimulate any production of IL-8 in neutrophil cultured for 18 h (**Figure 1F**), but abrogated IL-8 production induced by LPS at 2 h of culture (**Figure 1G**), suggesting that an increased osmolarity and not high sodium mediates the early suppression of IL-8 production triggered by LPS.

Considering previous studies showing that high salt promotes the activation of macrophages into a proinflammatory M1 profile *via* p38 MAPK-dependent signaling (6), we analyzed the involvement of this pathway in the stimulation of IL-8 production by high salt, by using two p38 MAPK inhibitors; SB202190 and SB203580. Both compounds markedly inhibited the production of IL-8 by high salt-treated neutrophils (**Figure 1H**). We then studied whether high salt might also be able to modulate the production of other cytokines by neutrophils. High salt did not induce the production of IL-1α, IL-6, IL-10 or TNF-α, but markedly enhanced the production of TNF-α, but not other cytokines, by LPS-primed cells (**Figure 1I**). Kinetic studies showed that the synergism exerted by high salt and LPS in the production of TNF-α required long incubation periods, and it was not evident before 8 h after stimulation (**Figure 1J**).

**Figure 2:**
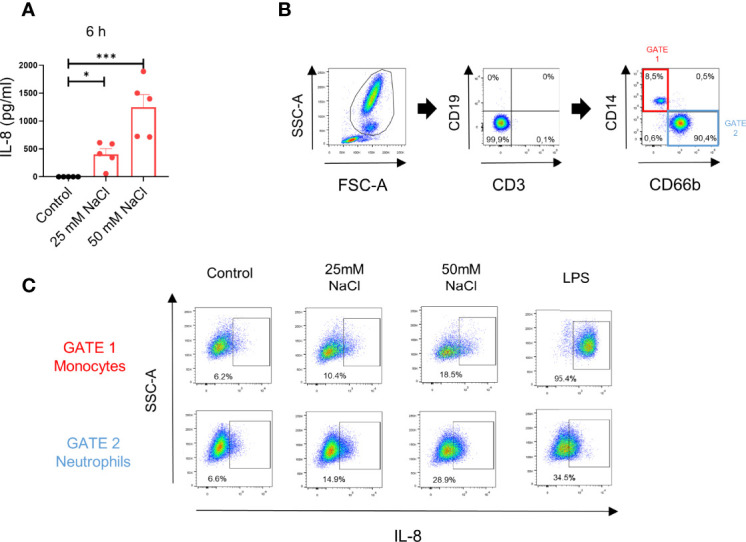
High salt induces IL-8 production in whole blood samples. **(A)** Whole blood samples (200 μl) supplemented or not with NaCl (25 mM and 50 mM) were incubated for 6 h at 37°C. Then, IL-8 in the plasma fraction was evaluated by ELISA. Results are expressed as the mean ± SE from 5 experiments. **(B)** Gating strategy used to evaluate the production of IL-8 by neutrophils and monocytes in whole blood samples. **(C)** Whole blood samples (200 μl) supplemented or not with NaCl (25 mM or 50 mM) or LPS (100 ng/ml) were cultured for 8 h (the last 4 h of incubation was performed in the presence of the Golgi transport inhibitor BFA 10 μg/ml), and IL-8 production was analyzed by intracellular staining and flow cytometry in the gate of neutrophils and monocytes. A representative experiment (n=3) is shown. *P <.05, and ***P <.001.

Consistent with data in **Figure 1**, assays performed in whole blood samples showed that high salt induced IL-8 production when evaluated by ELISA at 6 h of culture in the plasma fraction of blood samples (**Figure 2A**). We then analyzed the contribution of neutrophils and monocytes in the production of IL-8 observed in whole blood samples by intracellular staining and flow cytometry. The gating strategy is shown in **Figure 2B**. We found that both, neutrophils and monocytes contribute in a similar fashion to the production of IL-8 induced by high salt. By contrast, on a per cell basis, monocytes appear to produce higher levels of IL-8 than neutrophils, upon LPS stimulation (**Figure 2C**).

### High Salt Modulates the Production of Reactive Oxygen Species (ROS) by Neutrophils

**Figure 3:**
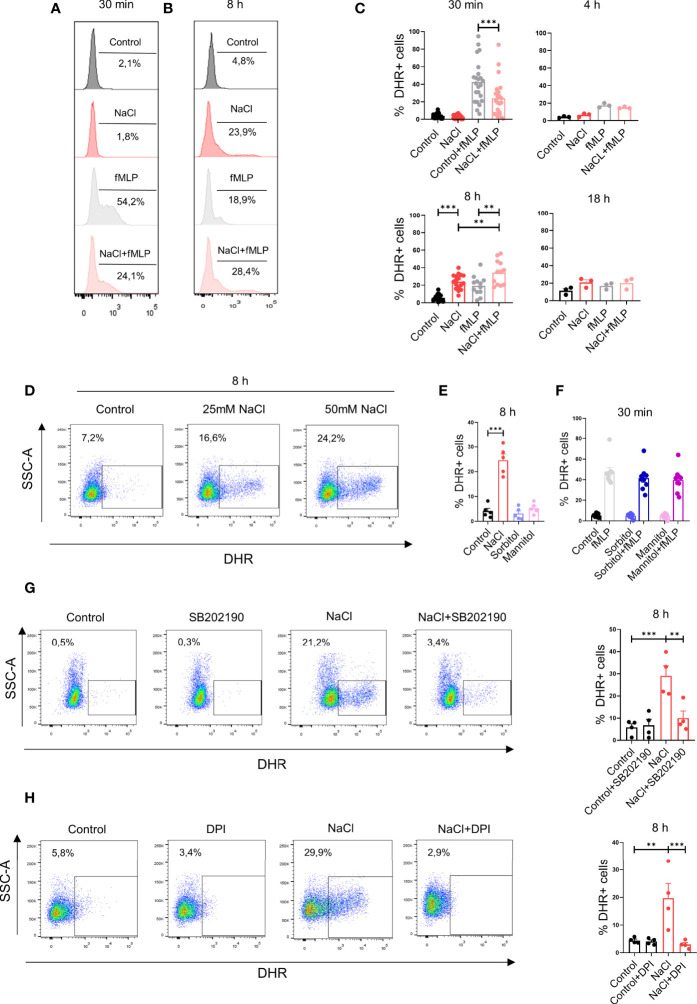
High salt modulates hydrogen peroxide production by neutrophils. **(A, B)** Neutrophils (2 x 10^6^/ml) were incubated at 37°C for 30 min **(A)** or 8 h **(B)** in culture medium supplemented, or not, with NaCl (50 mM). Cells were then labeled with dihydrorhodamine-123 and stimulated, or not, with fMLP (0.5 μM). After 20 min of incubation at 37°C, production of hydrogen peroxide was measured by flow cytometry. Representative experiments are shown. **(C)** Production of hydrogen peroxide was evaluated by flow cytometry in neutrophils cultured for different times with or without NaCl (50 mM), fMLP (0.5 μM) or NaCl (50 mM) plus fMLP (0.5 μM). Results are expressed as the mean ± SE from 14, 3, 17, and 3 experiments, for time points of 30 min, 4 h, 8 h and 18 h, respectively. **(D)** Neutrophils were incubated by 8 h in culture medium supplemented with 25 or 50 mM NaCl and the production of hydrogen peroxide was then evaluated. A representative experiment is shown. **(E)** Neutrophils were incubated for 8 h in culture medium supplemented with 50 mM NaCl, Sorbitol or Mannitol (100 mM) (final osmolarity of the culture) supplemented medium = 400 mOsm). Then, the production of hydrogen peroxide was evaluated. Results are expressed as the mean ± SE of 5 experiments. **(F)** Neutrophils were cultured for 30 min in culture medium supplemented, or not, with sorbitol or mannitol (100 mM) and/or fMLP (0.5 μM). Then, the production of hydrogen peroxide was evaluated. Results are expressed as the mean ± SE from 9 experiments. **(G**, **H)** Production of hydrogen peroxide was evaluated by flow cytometry in neutrophils cultured for 8 h in culture medium supplemented with 50 mM NaCl, in the absence or presence of the p38 MAPK inhibitor SB202190 (10 μM) **(G)** or the NADPH oxidase inhibitor diphenyleneiodonium (DPI, 10 uM) **(H)**. Representative experiments and the mean ± SE from 4 experiments are shown. **P <.01, and ***P<0.01.

We then analyzed the influence exerted by high salt on the production of ROS. Neutrophils were incubated for 30 min, 4 h, 8 h or 18 h in culture medium supplemented, or not, with 50mM NaCl and then they were treated, or not with fMLP. Exposure of neutrophils for short times to high salt (i.e., 30 min) did not induce the production of ROS while inhibited ROS production induced by fMLP. Consistent with the observations made for IL-8 production, we found that exposure of neutrophils for 8 h to high salt induced per se the production of ROS while increased ROS production assessed in the presence of fMLP (**Figures 3A–C**). **Figure 3D** compares the production of ROS induced by 25 mM and 50 mM NaCl, at 8 h of culture. Increasing osmolarity by sorbitol or mannitol neither stimulated ROS production in neutrophil cultured for 8 h (**Figure 3E**) nor inhibited ROS production induced by fMLP when evaluated after 30 min of culture (**Figure 3F**). This suggests that high salt, and not high osmolarity, was responsible for the modulation of ROS production by neutrophils. Consistent with the observations made for IL-8 production, we found that the inhibitor of p38MAPK SB202190 significantly suppressed ROS production induced by high salt (**Figure 3G**). As expected, the respiratory burst inhibitor diphenyleneiodonium (DPI) almost completely suppressed ROS production (**Figure 3H**).

### Effects Induced by High Salt on Neutrophil Degranulation, Phagocytosis and Apoptosis

**Figure 4:**
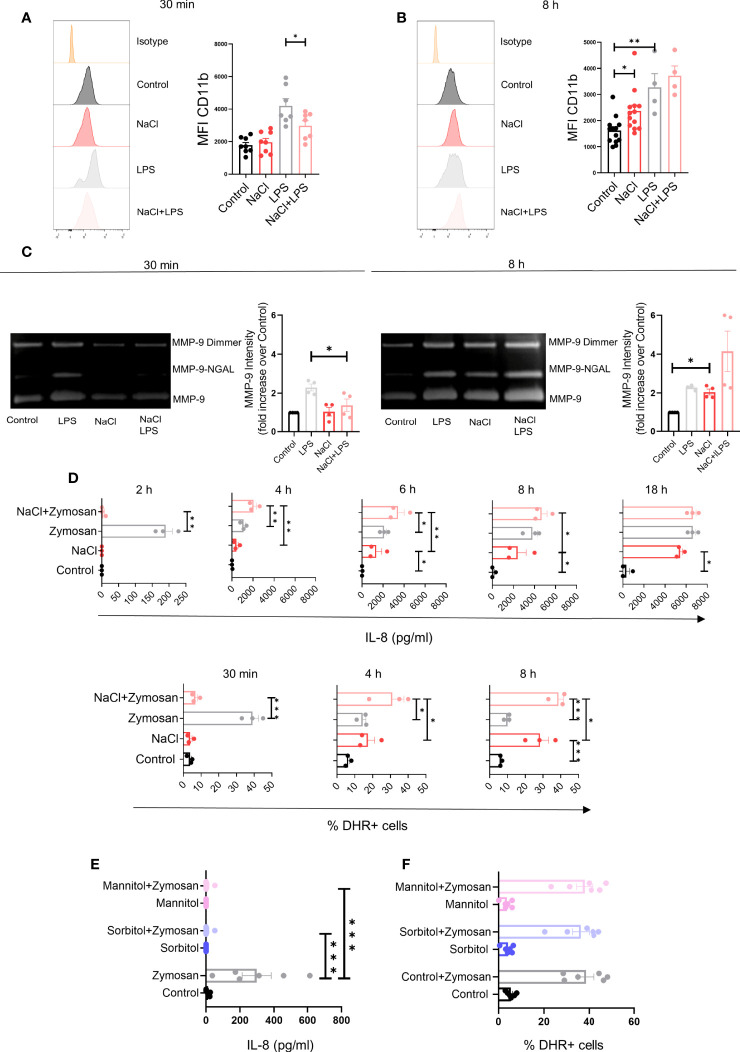
Effect of high salt on neutrophil degranulation induced by LPS and the production of IL-8 and hydrogen peroxide induced by Zymosan. **(A**, **B)** Neutrophils (2 x 10^6^/ml) were incubated at 37°C for 30 min **(A)** or 8 h **(B)** in culture medium supplemented, or not, with NaCl (50 mM) and/or LPS (100 ug/ml), and the expression of CD11b was then analyzed by flow cytometry. Representative experiments and the mean ± SE from 4-13 experiments are shown. **(C)** Neutrophils (5 x 10^6^/ml) were incubated at 37°C for 30 min or 8 h in culture medium supplemented or not with NaCl (50 mM) and/or LPS (100 ng/ml). Supernatants were then harvested and gelatinase activity was assessed as described under Materials and Methods. A representative experiment and the mean ± SE of 4 experiments are shown. The results are expressed in relative units having assigned the value of 1 to controls (neutrophils cultured in control medium). **(D)** Neutrophils (2 x 10^6^/ml) were incubated at 37°C for different times in culture medium supplemented, or not with NaCl (50 mM) and/or Zymosan (50 μg/ml). Then, the presence of IL-8 in cell supernatants and the production of hydrogen peroxide were evaluated by ELISA and flow cytometry, respectively. Results are expressed as the mean ± SE of 3 experiments. **(E**, **F)** Neutrophils were cultured for 30 min in culture medium supplemented, or not, with sorbitol or mannitol (100 mM) and/or Zymosan (50 μg/ml). Then, the presence of IL-8 in cell supernatants **(E)** and the production of hydrogen peroxide **(F)** was evaluated by ELISA and flow cytometry, respectively. Results are expressed as the mean ± SE from 6 experiments. *P <.05, **P <.01, and ***P <.001.

We also analyzed the influence of high salt on the expression of CD11b and the release of the matrix metalloproteinase-9 (MMP-9). Exposure of neutrophils to high salt for 30 min did not change the expression of CD11b but significantly inhibited its up-regulation induced by LPS (**Figure 4A**), while exposure to high salt for 8 h significantly increased CD11b expression in cells cultured in the absence or LPS, but not in the presence of LPS (**Figure 4B**). Additional experiments were performed to evaluate the release of the matrix metalloproteinase-9 (MMP-9), a potent angiogenic mediator (33), by SDS-PAGE. As previously described, MMP-9 released by neutrophils appears in three distinct protein bands; a homodimer of MMP-9, a heterodimer containing MMP-9 together with human neutrophil gelatinase-associated lipocalin (NGAL), and the monomeric form of MMP-9 (34). When evaluated after 30 min of exposure to high salt, an inhibition in the release of MMP-9 induced by LPS was observed (**Figure 4C**, left panels). By contrast, when evaluated after 8 h of exposure to high salt, we observed an increased release of MMP-9 in cultures performed in the absence of LPS, but not in those performed in the presence of LPS (**Figure 4C**, right panels).

Since neutrophils play a critical role not only in anti-bacterial immunity, but also in anti-fungal immunity, we analyzed the impact of high salt on neutrophil activation induced by Zymosan, a major component of the fungal cell wall (35). Consistent with the observations described in **Figures 1**, **3** for neutrophils stimulated with LPS or fMLP, we found that high salt promoted an early inhibition on the release of IL-8 and the production of hydrogen peroxide induced by Zymosan, followed by the stimulation of both responses at later time points (**Figure 4D**). Also consistent with the observations described above in neutrophils stimulated by LPS or fMLP, we found that short term exposure to increasing values of osmolarity by the addition of sorbitol or mannitol (100 mM) to the culture medium, abrogated IL-8 production without modifying ROS production in neutrophils stimulated by Zymosan (**Figures 4E, F**).

**Figure 5:**
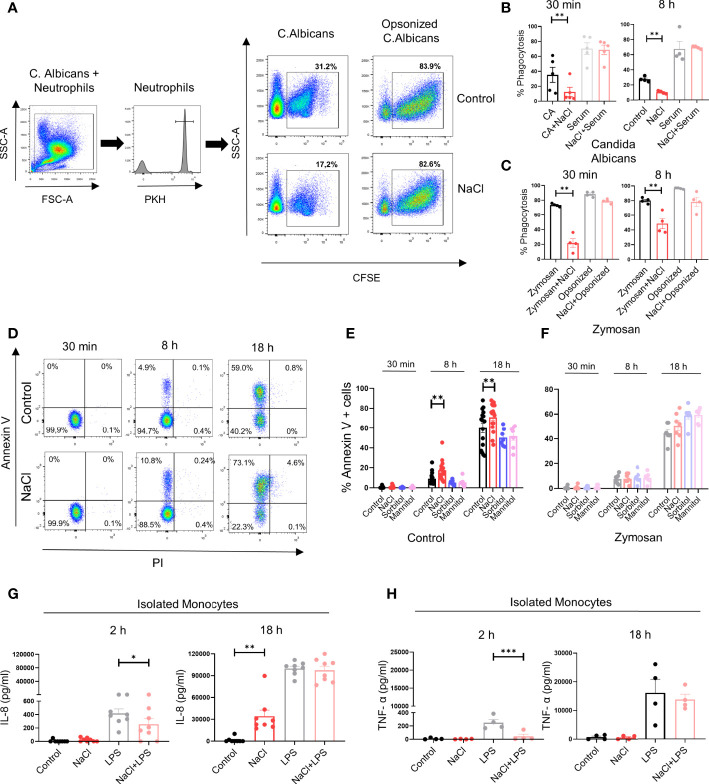
Effect of high salt on neutrophil phagocytosis and the production of IL-8 and TNF-α by monocytes. **(A)** Neutrophils were labeled with PKH26 and incubated (2 x 10^6^ neutrophils/ml) for 30 min in culture medium supplemented, or not, with NaCl (50 mM). Then, CFSE-labeled yeast (*C.albicans*) were added (neutrophil/yeast ratio of 1:1), in the absence or presence of fresh autologous serum (10% v/v). After 40 min of incubation at 37°C, yeast phagocytosis was evaluated in the gate of neutrophils (PKH26 positive cells) by flow cytometry using trypan blue to quench the fluorescence of the yeast attached (but not internalized) by neutrophils. A representative experiment and the mean ± SE from 5 experiments are shown. **(B)** Phagocytosis of *C. albicans* was evaluated as described in A, in neutrophils preincubated for 8 h in culture medium supplemented, or not, with NaCl (50 mM). Results represent the mean ± SE of 4 experiments. **(C)** Neutrophils (2 x 10^6^ neutrophils/ml) were cultured for 30 min or 8 h in medium supplemented, or not, with NaCl (50 mM). Then, fluorescent-labeled Zymozan particles (50 μg/ml) were added. After 1 h of incubation at 37°C, phagocytosis of Zymosan particles was evaluated by flow cytometry. Results are expressed as the mean ± SE from 4 experiments. **(D)** Neutrophils (2 x 10^6^/ml) were incubated at 37°C for 30 min, 8 h or 18 h in medium supplemented, or not, with NaCl (50mM). Then cells were labeled with annexin-V FITC and propidium iodide, and apoptosis was evaluated by flow cytometry. A representative experiment is shown. **(E**, **F)** Neutrophils (2 x 10^6^/ml) were incubated at 37°C for 30 min, 8 h or 18 h in medium supplemented, or not, with NaCl (50mM), sorbitol or mannitol (100 m), in the absence **(E)** or presence of Zymosan **(F)**. Then cells were labeled with annexin-V FITC and propidium iodide, and apoptosis was evaluated by flow cytometry. Results are expressed as the mean ± SE from 7-17 experiments. **(G**, **H)** Isolated monocytes (1 x 10^6^/ml) were incubated at 37°C for 2 or 18 h in culture culture medium supplemented, or not, with NaCl (50 mM) and/or LPS (100 ng/ml). Then, the production of IL-8 **(G)** and TNF-α **(H)** was evaluated in cell supernatants by ELISA. The mean ± SE from 4-8 experiments are shown. *P <.05, **P <.01, and ***P <.001.

We then analyzed the impact of high salt on the phagocytosis of *C. albicans*. Phagocytosis assays were performed either in the absence or presence of autologous serum, used as a source of opsonins. Short-term exposure to high salt (30 min) significantly inhibited yeast phagocytosis in assays performed in the absence but not in the presence of autologous serum. A similar response was observed using neutrophils previously cultured for 8 h in high salt-supplemented medium (**Figures 5A, B**). Neutrophil phagocytosis was also evaluated using fluorescent-labeled Zymosan. Consistent with the observations made by using *C. albicans* we found that phagocytosis of unopsonized Zymosan particles was inhibited by high salt when evaluated at either 30 min or 8 h of culture, while no inhibition was observed using opsonized Zymosan particles (**Figure 5C**).

Finally, we analyzed whether high salt affected neutrophil survival. A low but significant increase in the rate of apoptosis was observed when it was evaluated either at 8 or 18 h of culture. Increasing osmolarity by sorbitol or mannitol did not modulate the neutrophil apoptotic rate (**Figures 5D, E**). Moreover, experiments performed with Zymosan-stimulated neutrophils showed that neither high salt nor sorbitol or mannitol modulated the rate of apoptosis (**Figure 5F**).

### High Salt Also Exerts a Biphasic Effect on IL-8 Production by Monocytes

We then analyze whether the biphasic effect induced by high salt on neutrophils was also observed in monocytes. To this aim, we studied the production of IL-8 and TNF-α. Consistent with the observations made in neutrophils, we found that exposure of monocytes to high salt for 2 h inhibited IL-8 production induced by LPS, while overnight culture in high-salt supplemented medium resulted in the induction of IL-8 production in the absence of LPS stimulation (**Figure 5G**). A significant inhibition of TNF-α production induced by LPS was also observed at 2 h of culture under high salt conditions. Contrasting with the observations made for IL-8, high salt was shown to be completely unable to trigger TNF-α production in the absence of LPS stimulation in overnight cultures (**Figure 5H**).

## Discussion

In this study we show that high salt appears to induce opposite effects on neutrophils depending on the exposure time. Culture of neutrophils under high salt conditions induced a short-term inhibition characterized by the lack of response to conventional agonists such as LPS, the chemotactic peptide fMLP or Zymosan. By contrast, exposure to high salt for longer periods (6-18 h) resulted in both the activation of neutrophil inflammatory responses and an increased response to conventional agonists. Assays under high salt conditions were carried out using culture medium supplemented with 50 mM NaCl, raising the possibility that the observed effects were induced by hyperosmotic stress. The unability of sorbitol and mannitol to exert any stimulatory effect on neutrophils suggests that hyperosmotic stress is not involved in the activation of neutrophil responses induced by high salt. Interestingly, sorbitol and mannitol markedly suppressed the production of IL-8 induced by conventional agonists when it was evaluated at early time points without affecting the production of ROS, thus suggesting that short term exposure to hyperosmotic stress selectively modulates different neutrophils functions.

Not all the neutrophil responses appear to be regulated in a biphasic mode depending on the exposure time to high salt. The ability of neutrophils to phagocyte unopsonized yeasts or Zymosan was shown to be inhibited even when it was assessed after long periods of incubation with high salt. Interestingly, no inhibition was observed using serum-opsonized yeasts or Zymosan, suggesting that the impact of high salt on phagocytosis strongly depends on the properties of the particle to be phagocytosed.

Previous studies have established that high salt induces the acquisition of an inflammatory profile by macrophages (20–22), promotes the development of TH17 responses (15, 19) and suppresses the function of regulatory T cells (16). All these studies suggest a contribution of innate and adaptive immune mechanisms in the development of inflammatory reactions in high salt environments. Interestingly, more recent work suggest that high salt might also induce anti-inflammatory mechanisms. Matthias and coworkers (36) have recently reported a context-dependent, dichotomous role for high salt in determining TH17 cell function and pathogenicity. The authors found that T cell stimulation in a proinflammatory and TGF-β-low cytokine environment results in the acquisition of a pathogenic profile by TH17. By contrast, the development of TH17 under non-inflammatory conditions results in the acquisition of an anti-inflammatory signature. High salt has also shown to mediate anti-inflammatory mechanisms *via* endocrine pathways. Na SY and coworkers (37) have reported that high salt consumption increased blood serum levels of corticosterone which results in an enhanced expression of tight junction molecules on the brain endothelial cells promoting the tightening of the blood brain barrier thereby controlling the entry of inflammatory T cells into the central nervous system.

Contrasting results have been published regarding the effects induced by high salt on neutrophil function and pathogenicity. Most of previous published reports have shown that high salt suppresses neutrophil function. In fact, high salt has shown to inhibit the up-regulation of CD11b/CD18 expression induced by LPS (23), the chemotactic response (25), the bactericidal activity (27–29), the exocytosis of the content of the different neutrophil granules as well as the release of ROS and neutrophil extracellular traps induced by different stimuli (24, 28, 29, 38). By contrast, it has been reported that high salt induces the release of neutrophil extracellular traps (30) and enhances host response to bacterial challenge in experimental models, by increasing receptor-independent production of ROS by neutrophils (26). The reasons for these contradictory results are unclear but might reflect the use of different experimental conditions. In fact, opposite effects on neutrophil function were reported depending on the timing of exposure to high salt relative to other stimuli. Prior exposure to high salt has shown to suppress the release of elastase induced by fMLP stimulation while it was enhanced when high salt was added after fMLP stimulation (39). A similar contrasting effect was observed when the production of ROS was evaluated in neutrophils stimulated by platelet activating factor (PAF) plus fMLP (40). Our present results add a new level of complexity to the understanding of the effects induced by high salt on neutrophil function, suggesting that the exposure time represents a critical variable that might determine the suppression or stimulation of different neutrophil functions.

Our observations show that exposure of neutrophils to high salt for long periods (6-18 h) triggers different responses without the addition of any other stimulus. Interestingly, the levels of IL-8 production induced by high salt were comparable to those induced by LPS. Two previous studies have analyzed the impact of high salt on neutrophil production of IL-8. Hatanaka and coworkers (31) reported that high salt suppressed IL-8 production by either unstimulated or LPS-stimulated neutrophils and mononuclear cells. The reasons underlying the discrepancy between our results and those reported by Hatanata and coworkers are not clear, since similar experimental conditions were used in both studies. On the other hand, and consistent with our observations, Krampert and coworkers (29) recently reported that high salt induced IL-8 production and boosted IL-8 production of neutrophils infected with *E.coli*. However, contrasting with our results, Krampert and coworkers observed that high salt boosted IL-8 production after a short incubation period, i.e., 1.5 h, time at which we observed a profound inhibition in the production of IL-8 triggered by LPS. The reasons for these discrepant results are not clear but they could be related to the use of different stimuli in each case; an infectious bacteria or LPS.

Of note, we also found that high salt efficiently stimulated TNF-α production by isolated neutrophils primed with LPS. Contrasting with the observations made for IL-8, no production of TNF-α was observed for neutrophils stimulated only by high salt. The mechanisms underlying the ability of high salt to differentially regulate the production of IL-8 and TNF-α remain to be established. We also analyzed the ability of high salt to trigger IL-8 production in assays performed in whole blood samples. Flow cytometric analysis showed that the addition of either high salt or LPS induced the production of IL-8 by neutrophils or monocytes. Interestingly, we found that both cell types produced similar amounts of IL-8 in response to high salt. This was unexpected considering that neutrophils usually produce much lower levels of cytokines compared to monocytes (41). Interestingly, we also observed that short term exposure of isolated monocytes to high salt also suppressed IL-8 and TNF-α production induced by LPS while culture for longer periods (i.e., overnight cultures) resulted in the induction of IL-8, but not TNF-α, in the absence of LPS-stimulation.

Despite the numerous studies published directed to clarify the immunoregulatory properties of high salt environments, the mechanisms through which immune cells sense increased sodium concentrations in the extracellular medium remain largely elusive. Pioneering observations published 25 years ago by Rosette C and Karin M (42) showed that osmotic stress induced by high concentrations of sorbitol results in a ligand-independent clustering and activation of cell surface receptors for epidermal growth factor (EGF), TNF-α, and IL-1 in HeLa cells. All of these receptors are expressed by neutrophils (43–45). However, this mechanism does not appear to explain the stimulatory effect induced by high salt on neutrophils since neither mannitol nor sorbitol induced any stimulatory effect, suggesting that high salt and not high osmolarity is responsible for the induction of neutrophil activation. Neutrophils express a number of Na+ transport proteins that might mediate the increase in intracellular Na+ concentrations when placed in high salt environments. Interestingly, previous studies have shown that not only the influx of Ca2+ but also Na+ influx play a major role in neutrophil activation induced by fMLP (46, 47). While the mechanisms through which neutrophils senses the presence of high salt concentrations remain to be elucidated, consistent with previous studies performed in macrophages (20, 21) and CD4+ T cells (15, 19), we found that the acquisition of an inflammatory signature by neutrophils cultured under high salt conditions proceed through a p38 MAPK-dependent pathway.

In brief, our results show that the modulation of neutrophil function by high salt is strongly dependent on the exposure time. Considering that neutrophils do not infiltrate peripheral tissues in the absence of microbial or danger stimuli, the early inhibition of neutrophil function in response to sodium-rich environments might contribute to minimizing tissue injury until their arrival to the infectious focus.

## Data Availability Statement

The original contributions presented in the study are included in the article/supplementary material. Further inquiries can be directed to the corresponding author.

## Author Contributions

IM and LB designed and conducted experiments, collected data and performed data analysis. CM and PG conducted flow cytometry studies and designed experiments. AT and MC cultivated C. Albicans. FD conducted confocal microscopy studies and designed experiments. JG was responsible for overall experimental design and supervision of studies. All authors contributed to the article and approved the submitted version.

## Funding

This work was supported by grants from the Fondo Nacional para la Investigación Científica y Tecnológica (PICT 2017-1616 and PICT 2018–02844 to JG) and the Universidad de Buenos Aires (20020170100573BA to JG).

## Conflict of Interest

The authors declare that the research was conducted in the absence of any commercial or financial relationships that could be construed as a potential conflict of interest.

## Publisher’s Note

All claims expressed in this article are solely those of the authors and do not necessarily represent those of their affiliated organizations, or those of the publisher, the editors and the reviewers. Any product that may be evaluated in this article, or claim that may be made by its manufacturer, is not guaranteed or endorsed by the publisher.
